# IL22 Regulates Human Urothelial Cell Sensory and Innate Functions through Modulation of the Acetylcholine Response, Immunoregulatory Cytokines and Antimicrobial Peptides: Assessment of an *In Vitro* Model

**DOI:** 10.1371/journal.pone.0111375

**Published:** 2014-10-29

**Authors:** Phong T. Le, Meghan M. Pearce, Shubin Zhang, Edward M. Campbell, Cynthia S. Fok, Elizabeth R. Mueller, Cynthia A. Brincat, Alan J. Wolfe, Linda Brubaker

**Affiliations:** 1 Department of Microbiology and Immunology, Stritch School of Medicine, Loyola University Chicago, Maywood, Illinois, United States of America; 2 Department of Obstetrics and Gynecology, Stritch School of Medicine, Loyola University Chicago, Maywood, Illinois, United States of America; 3 University of Minnesota, Department of Urology, Minneapolis, Minnesota, United States of America; University of Missouri-Kansas City, United States of America

## Abstract

Human urinary disorders are generally studied in rodent models due to limitations of functional *in vitro* culture models of primary human urothelial cells (HUCs). Current HUC culture models are often derived from immortalized cancer cell lines, which likely have functional characteristics differ from healthy human urothelium. Here, we described a simple explant culture technique to generate HUCs and assessed their *in vitro* functions. Using transmission electron microscopy, we assessed morphology and heterogeneity of the generated HUCs and characterized their intercellular membrane structural proteins relative to *ex vivo* urothelium tissue. We demonstrated that our cultured HUCs are free of fibroblasts. They are also heterogeneous, containing cells characteristic of both immature basal cells and mature superficial urothelial cells. The cultured HUCs expressed muscarinic receptors (MR1 and MR2), carnitine acetyltransferase (CarAT), immunoregulatory cytokines IL7, IL15, and IL23, as well as the chemokine CCL20. HUCs also expressed epithelial cell-specific molecules essential for forming intercellular structures that maintain the functional capacity to form the physiological barrier of the human bladder urothelium. A subset of HUCs, identified by the high expression of CD44, expressed the Toll-like receptor 4 (TLR4) along with its co-receptor CD14. We demonstrated that HUCs express, at the mRNA level, both forms of the IL22 receptor, the membrane-associated (IL22RA1) and the secreted soluble (IL22RA2) forms; in turn, IL22 inhibited expression of MR1 and induced expression of CarAT and two antimicrobial peptides (S100A9 and lipocalin-2). While the cellular sources of IL22 have yet to be identified, the HUC cytokine and chemokine profiles support the concept that IL22-producing cells are present in the human bladder mucosa tissue and that IL22 plays a regulatory role in HUC functions. Thus, the described explant technique is clearly capable of generating functional HUCs suitable for the study of human urinary tract disorders, including interactions between urothelium and IL22-producing cells.

## Introduction

*The urothelium* is the layer of stratified epithelial cells that lines the lumen of the bladder and urinary tract. Whereas the urothelium forms a physical barrier, it also performs other functions [1]. For example, it responds to diverse stimuli, including mechanical, chemical and biological signals. In response, the urothelium releases mediators that contribute to the maintenance of urothelium functions and homeostasis. While most current understanding of urothelium biology and physiopathology has been gained from studying animal models, less is known about the human urothelium, mainly due to limitations in establishing a functional primary human urothelial cell (HUC) culture system.

The human urothelium is organized into three distinct cell layers: the basal, intermediate and outermost superficial layer. The superficial layer consists of terminally differentiated umbrella cells, which establish and maintain the physical barrier function through intercellular tight junctions. The apical face of the umbrella cells is covered with a unique, asymmetric unit membrane called the uroplakin plaque (Uro P), in which the four members of the uroplakin protein (UP) family are assembled into two distinct heterodimer units [2], [3]. Umbrella cells also contain intracellular organelles, the discoid fusiform vesicles (DFVs); in response to mechanical stimuli, DFVs are incorporated into the cell membrane to increase bladder volume [4].

The urothelium of various species has been shown to express choline acetyltransferase (ChAT) and/or carnitine acetyltransferase (CarAT), enzymes that synthesize the neurotransmitter acetylcholine (Ach) [5], [6]. Urothelium also expresses cholinergic receptors, including the nicotinic and muscarinic receptors (MR) that mediate the Ach response [7], [8]. Of particularly interest is the functional discovery of two main groups of MR: the MR1 (which also includes MR3 and MR5) and MR2 (which also includes MR4) [6]–[8]. In the urothelium, expression of MR1 is predominantly found in basal cells, while MR2 expression is restricted to umbrella cells [8]. Thus, the urothelium is a non-neuronal source of Ach, which together with expression of MR, has been implicated in the development of certain lower urinary tract disorders, via establishment of an autocrine pathway [1], [9]. The modulation of this Ach response in human urothelium is not well understood.

Our recent work has convincingly demonstrated that a human urinary microbiota exists [10]–[12]. In other human mucosa tissues, the maintenance of organ-specific microbiota is regulated by innate and adaptive immune cells through production of various immunoregulatory cytokines and antimicrobial peptides (AMPs) that both control invasion by bacterial pathogens and potentially regulate microbial diversity [13]–[15]. Despite the significant disease burden of urinary tract infection, urinary incontinence, and other related urinary disorders, little is known about the immune regulation of the human bladder. Since interleukin 22 (IL22) is an important cytokine that regulates pathogen invasion in the gastrointestinal tract [16], [17], it is possible that IL22 plays a similar role in maintaining the human bladder microbiota.

Human urothelial cultures currently employed are typically bladder carcinoma cell lines, whose use limits our ability to describe normal urothelial cell functions. Thus, to advance our current understanding of human urothelium biology and physiopathology, a functional *in vitro* culture system that retains key aspects of the human urothelium is needed. Such aspects would include heterogeneity of uroepithelial cell populations, as well as the expression of critical molecules that emulates their functions *in vivo*.

Here, we describe a simple explant culture technique that readily generates human urothelial cell (HUC) cultures with functional characteristics. Using, these HUC cultures, we demonstrate for the first time that IL22 plays an important role in the maintenance of human urothelium function and provide evidence that HUCs potentially interact with immune cells that contribute to the maintenance of the human bladder microbiota.

## Results

### Primary cultures of Human Urothelial Cells (HUCs) established from bladder biopsy

**Figure 1:**
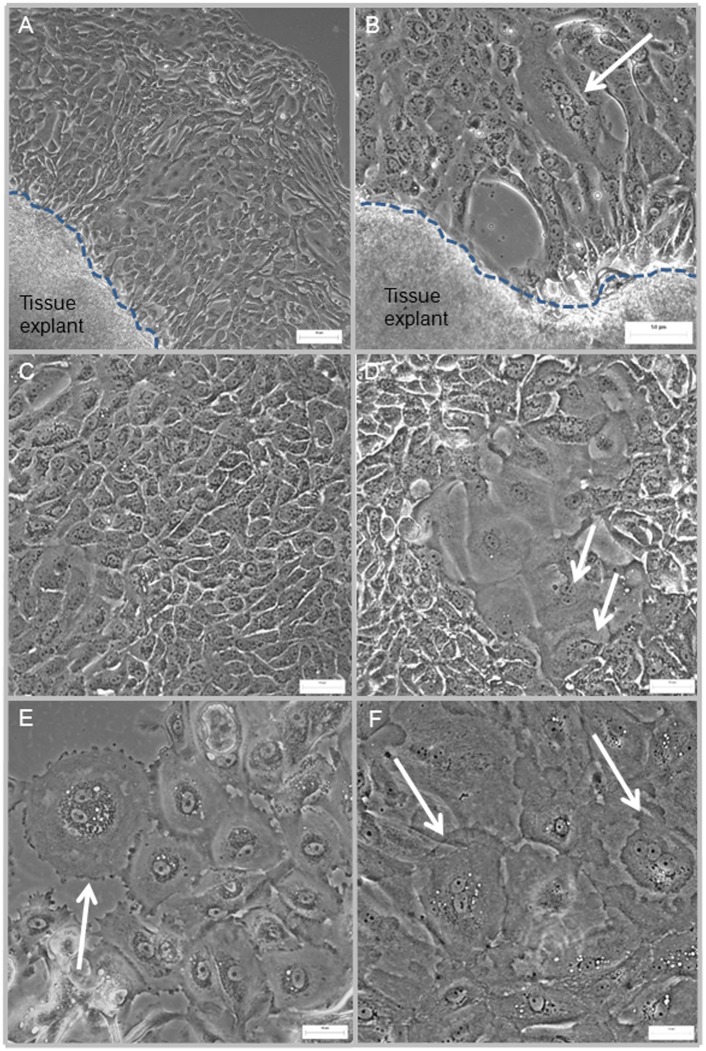
Cellular morphology of human urothelial cells (HUCs) generated from bladder tissue explant. (A, B) Cells growing out from the edge of two tissue explants obtained from two different biopsies; cells with typical coble-stone morphology were clearly identifiable. Arrow points to a larger cell (60–150 µm) with four nuclei and prominent nucleolus. (C, D) cells with uniform size and morphology (25–40 µm) surround a cluster of larger cells (70–100 µm); several are bi-nucleated (arrows). (E, F) clusters of larger cells (80–150 µm), many of these cells are bi-nucleated (arrows). The dotted lines demarcate the edge of the tissue explants. Scale bar = 50 µm.

Using an explant culture technique previously established in our laboratory to generate primary cultures of human thymic epithelial cells [18], we generated HUC cultures from human bladder urothelial biopsies obtained from consenting surgical patients following human subjects institutional review board approval. The explant technique generated HUC growth from bladder biopsies in most (80–90%) of the tissue explants. Cells with epithelial cell morphology appeared at the edge of the tissue explants within 1 week in culture (**Fig. 1A, B**). Upon removal of the tissue explants (around day 10–12), clusters of the proliferating cells further expanded into growth areas of approximately 5–10 cm^2^. Within this growth area, the cells appeared to be well interconnected with cobble-stone morphology, characteristic of epithelial cells in culture (**Fig. 1C, D**). Among the predominant cells with uniform size (25–40 µm in diameter), we observed clusters of larger bi-nucleated cells (70–150 µm in diameter) (**Fig. 1C–F**). Cells at this stage could be sub-cultured once; additional passages did not result in further expansion of the HUC cultures.

### Phenotypes of primary cultures of HUCs

**Figure 2:**
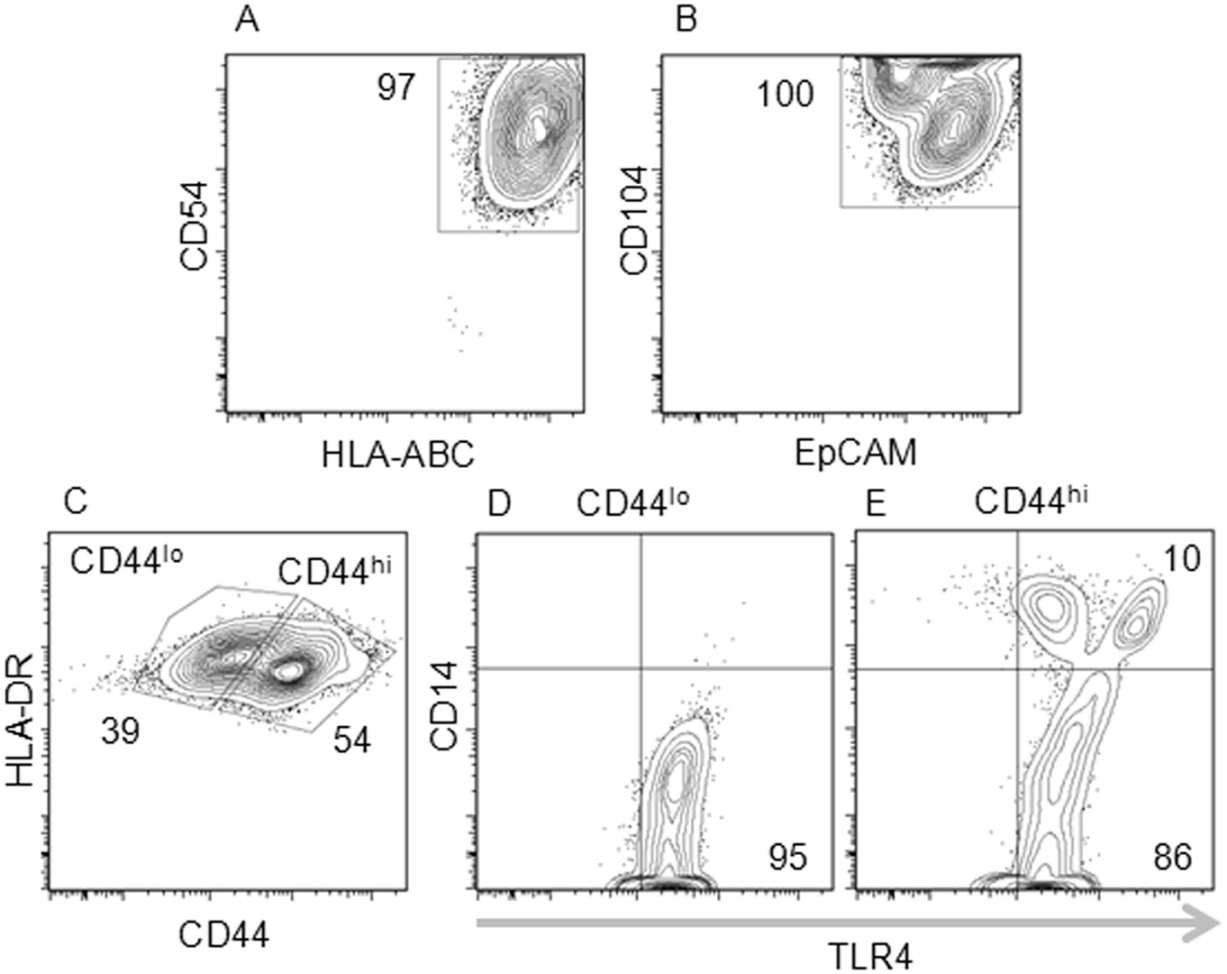
Flow cytometric analysis of primary HUCs. Cells were stained with eight fluorochrome-conjugated monoclonal antibodies direct against cell surface makers. Cells were analyzed for the expression of: (A) HLA-ABC and CD54 (ICAM-1); (B) CD104 and EpCAM; (C) HLA-DR, and CD44– expression of CD44 shows two distinct populations of HUCs: CD44^lo^ and CD44^hi^; (D, E) TLR and CD14. Representative data from 3 experiments performed with HUCs generated from 3 independent bladder biopsies.

To characterize the established HUCs, we examined expression of epithelial cell-specific and other cell surface markers, using multi-color flow cytometric analysis. We first determined that HUCs uniformly express human major histocompatibility complex class I HLA-ABC, verifying that they were derived from human tissues (**Fig. 2A**). These HLA-ABC^pos^ HUCs expressed the intercellular adhesion molecule-1 (ICAM-1, CD54) (**Fig. 2A**). They also expressed the epithelial cell adhesion molecule (EpCAM, CD326) and integrin-β4 (CD104), two specific markers for epithelial cells (**Fig. 2B**). HUCs were negative for human major histocompatibility complex class II HLA-DR, suggesting that expression of HLA-DR *in vitro* requires further stimulation (**Fig. 2C**). Based on the levels of the cell surface expression of CD44, a hyaluronan binding protein, HUCs could be divided into two distinct populations (**Fig. 2C**). While both populations expressed the Toll-like receptor 4 (TLR4), only the HUC population with high expression of CD44 expressed the TLR4 co-receptor CD14 (**Fig. 2C–E**).

### Ultrastructure characteristics of primary HUCs

**Figure 3:**
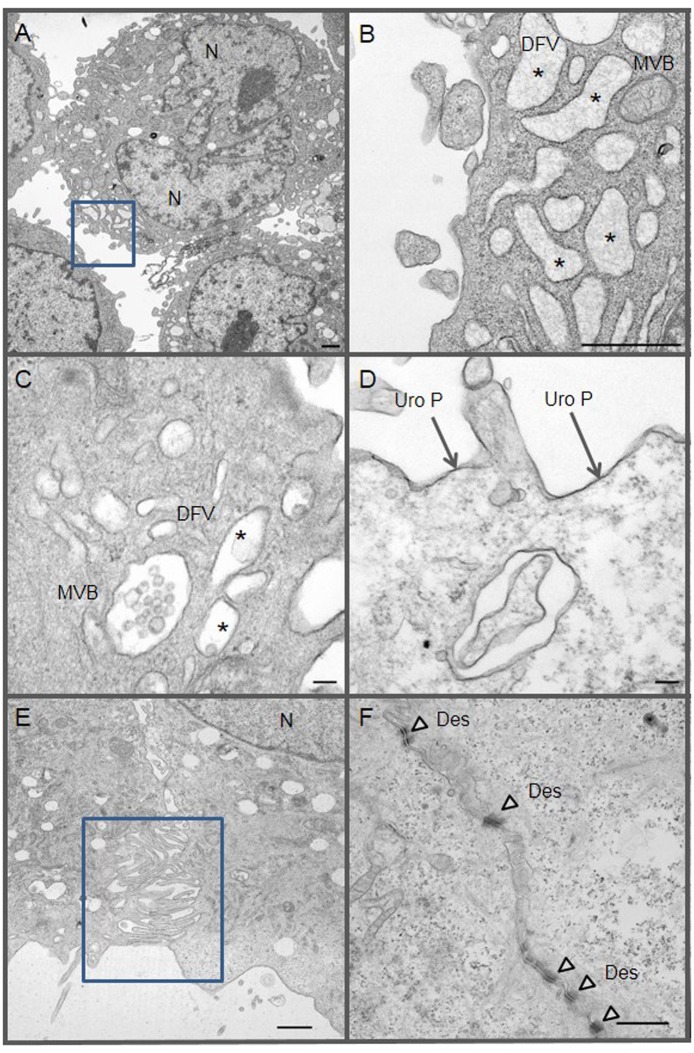
Ultrastructures of primary HUCs. Cultured HUCs were analyzed by TEM. (A, B) a HUC with two nuclei (N) and many discoid fusiform vesicles (DVF) - *denotes lumens of DFV; B is a higher magnification of the square area identified in A - MVB denotes multivesicular body; (C) another example of MVB and DFV in a HUC; (D) presence of uroplakins plaques (Uro P, dark line) on the cell membrane of an umbrella cell; (E) formation of lateral interdigitation between two adjacent HUCs, a higher magnification of the identified was shown in F; (F) presence of numerous desmosomes (Des, arrowheads) within a well-formed lateral interdigitation of two adjacent HUCs. Bar scale is equal to 1 µm (in A, B, E and F) and 0.1 µm (in C and D).

**Figure 4:**
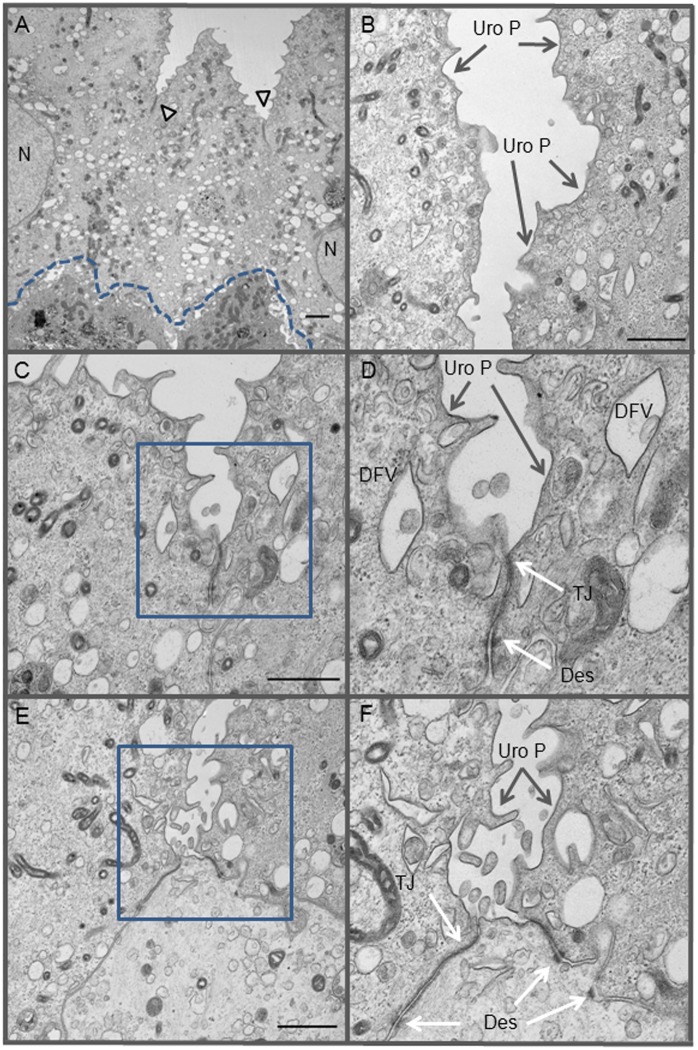
Ultrastructures of *ex vivo* human bladder urothelium. Bladder tissue biopsy was immediately processed for TEM study. (A) Surface umbrella cells with numerous DFVs. Arrowheads point to tight junctions (TJ) between two adjacent umbrella cells; dotted line demarcates junction between the upper umbrella cells and the underlying urothelial cells. (B) A higher magnification photograph of umbrella cells showing DVFs and uroplakins plaques (Uro P), which appear as darker lines associated with the cell membrane. (C, E) Higher magnification of TJ structures shown in A. (E, F) enlargement of areas shown in C and E (square boxes) showing DFVs, Uro P, TJ and desmosomes (Des). Bar scale = 1 µm.

The presence of larger, bi-nucleated cells suggested that these cells are the mature superficial umbrella cells that line the adult human bladder lumen (**Fig. 1B, D–F**). To determine if our culture system generated umbrella cells, we performed transmission electron microscopy (TEM) studies (**Fig. 3A–F**), seeking evidence of two structures that are unique to umbrella cells: the discoid fusiform vesicle (DVF) and the uroplakin plaque (Uro P). In our primary HUCs, we identified cells with numerous DVFs and Uro Ps (**Fig. 3D**); identical structures were also observed in umbrella cells that were located in the outer most layer of *ex vivo* bladder urothelium (**Fig. 4B–D**). We also observed multivesicular bodies (MVBs) with exosomes in cultured HUCs (**Fig. 3B, C**). In culture, adjacent HUCs formed lateral interdigitations (**Fig. 3E**), which increase surface connection between two cells. They also formed desmosomes (Des), which further strengthen cell-cell connections (**Fig. 3F**). These ultrastructures were similarly observed in the *ex vivo* human bladder biopsy (**Fig. 4**).

**Figure 5:**
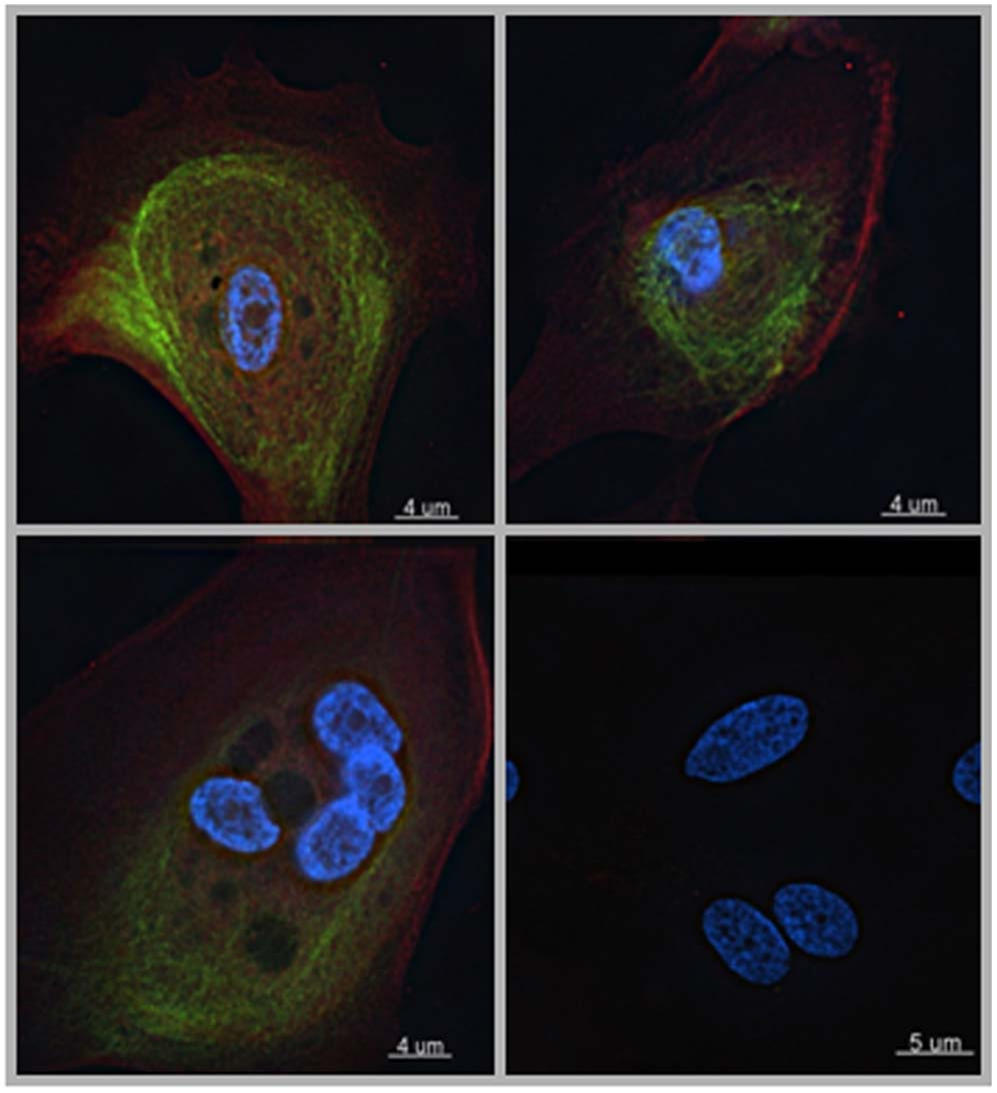
Immunofluorescent staining of cultured HUCs. HUCs were fixed and double stained with anti-uroplakins (PE, Red) and keratin-20 (FITC, green). DAPI (blue) was used to stain the nucleus. Lower right panel is a negative control stained with mouse and rabbit IgG. Representative images from 3 experiments from 3 independent biopsies.

Thus, both cellular morphology and ultrastructure characteristics indicate the presence of umbrella cells in our HUC cultures. To further confirm that our culture conditions generated umbrella cells from human bladder biopsies, we performed indirect immunofluorescent staining, using anti-uroplakin and anti-keratin 20 antibodies. We detected cells positive for both uroplakins (red) and keratin 20 (green), indicative of umbrella cells (**Fig. 5**).

**Figure 6:**
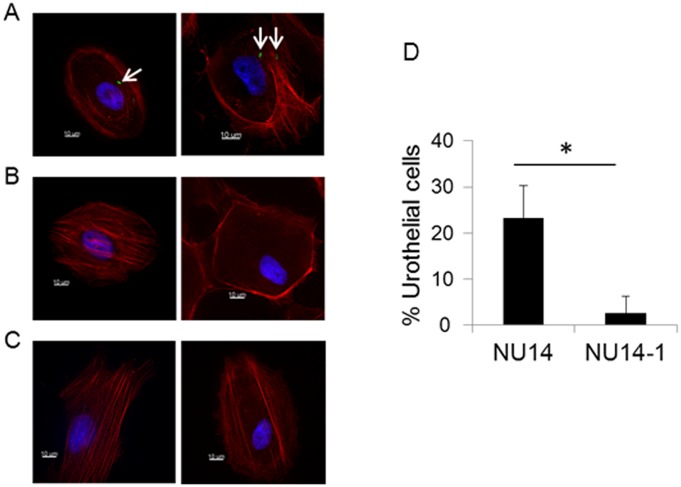
Uropathogenic *E. coli* strain NU14 adheres to HUCs. HUCs were exposed to NU14 (A), the isogenic *fimH* mutant NU14-1 (B) or media alone (C). After 2 h, the HUCs were washed and stained for the presence of adherent bacteria (green). Host cell actin (red) and nuclei (blue) were counterstained. Merged images show NU14 (A, arrows) adhering to urothelial cells. The percentage of urothelial cells with adherent bacteria was quantified (D) by scoring at least 200 urothelial cells from random fields of view for the presence of adherent bacteria. The percentage represents the number of urothelial cells with adherent bacteria divided by the total number of urothelial cells examined. Data are representative of at least three (A–C) or two (D) independent experiments performed with cells derived from independent biopsies. Between experiments, the average percentage of urothelial cells with adherent NU14 ranged from 8 to 23%. The errors bars represent the standard deviation of two coverslips per strain per experiment. Scale bars, 10 µm. *Indicates p<0.05, Student’s t-test.

Since umbrella cells line the luminal surface of the bladder, they are the most likely targets for interaction with uropathogenic *Escherichia coli* (UPEC). It has been demonstrated previously that UPEC utilize type I pili to adhere to cell surface uroplakins expressed by umbrella cells [19]–[21]. To examine whether UPEC adhere to umbrella cells present in our primary HUCs, we incubated HUCs in the presence of the UPEC strain NU14 for 2 h, removed non-adherent bacteria through PBS washes and assessed for bacterial adherence via microscopy. NU14 adhered to HUCs (**Fig. 6A, D**). This interaction was mediated by type I pili, as the *fimH* mutant (NU14-1) exhibited decreased adherence compared to wild-type (**Fig. 6B, D**).

### Functional analysis of primary HUCs

To evaluate potential functions of cultured HUCs, we assessed expression of various genes that have been implicated in urothelium physiopathology. We also assessed expression of cytokines or chemokines that potentially affect urothelium homeostasis and/or modulate immune cell functions within the human bladder mucosa tissue. By quantitative RT-PCR (qRT-PCR), we determined that primary HUCs express CarAT (**Table 1**), an enzyme that catalyzes the synthesis of acetylcholine. In contrast, expression of choline acetyltransferase (ChAT), which catalyzes acetylcholine synthesis in neurons, was not detectable in our HUC cultures (data not shown). The cultured HUCs expressed predominantly the muscarinic acetylcholine receptor group 1 (MR1); MR2 also was detectable, albeit at a very low level; perhaps, the data reflect the low percentage of umbrella cells in the cultures (**Table 1**).

**Table 1:** Expression of cytokines, cytokine receptors, chemokines in primary HUCs (#Number of copies/µg total RNA).

Genes	Experiment 1	Experiment 2	Experiment 3
MR1	2.7×10^5^ (2×10^4^)#	1.3×10^4^ (6.8×10^3^)	8.4×10^4^ (1.8×10^4^)
MR2	2.3×10^3^ (2×10^2^)	1.7×10^3^ (2.4×10^2^)	0.3×10^3^ (3)
CarAT	8.1×10^5^ (1×10^5^)	2.9×10^5^ (1.6×10^4^)	1.6×10^5^ (1.0×10^4^)
IL7	1.2×10^6^ (1.7×10^5^)	4.1×10^6^ (2.0×10^5^)	3.1×10^5^ (4.8×10^4^)
IL15	1.2×10^4^ (3.2×10^3^)	2.6×10^5^ (4.0×10^4^)	6.4×10^3^ (1.3×10^3^)
IL23	6.4×10^3^ (1.3×10^3^)	1.2×10^4^ (9.2×10^2^)	7.1×10^3^ (1.1×10^3^)
CCL20	6.4×10^5^ (1.7×10^5^)	4.5×10^6^ (1.2×10^5^)	1.5×10^6^ (7.8×10 ^5^)
IL22RA1	4.2×10^5^ (2.1×10^4^)	1.3×10^6^ (1.4×10^5^)	1.1×10^5^ (1×10^4^)
IL22RA2	8.1×10^2^ (2.4×10^2^)	5.4×10^3^ (9.0×10^2^)	0.6×10^3^ (50)

Data were from three independent cultures of HUCs established from three different human bladder biopsies. Transcript levels were determined by qRT-PCR.

# Values represent the average of triplicates; numbers in parentheses are standard errors of the mean.

HUCs also expressed interleukins (IL) 7, 15 and 23, and the chemokine CCL20 (**Table 1**). IL7 and IL15 play important roles in T cell functions and homeostasis [22], while CCL20 plays a role in regulating mucosa lymphoid tissue formation by attracting T cells and dendritic cells toward and into urothelium [23]. IL23 is required for regulating IL22 expression [17].

### IL22 and HUC functions

**Figure 7:**
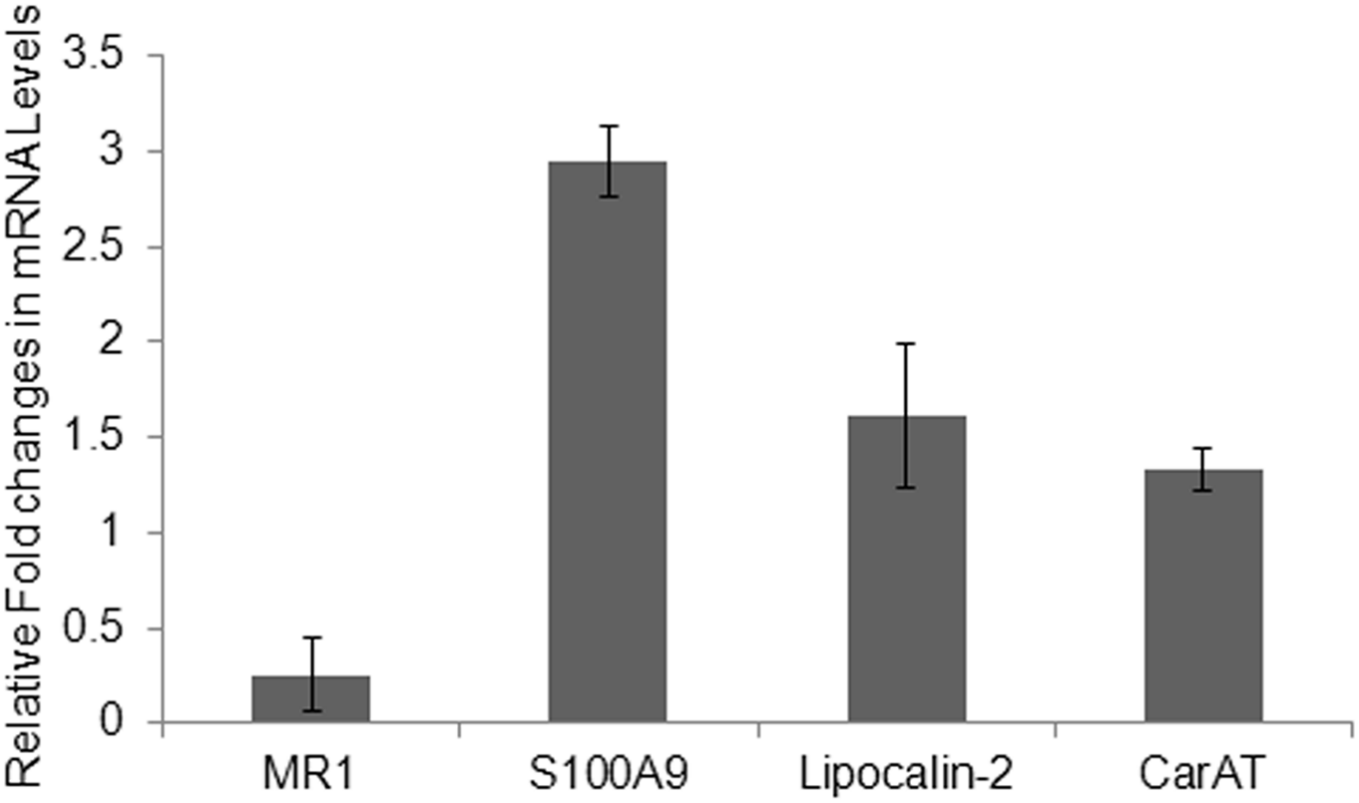
Effect of IL22 on the expression of MR1, S100A9, lipocalin-2, and CarAT by primary HUCs. Transcript levels were measured by qRT-PCR and expressed as fold change of treated versus untreated HUC cultures. Values represent the average of two independent experiments performed with two different HUC cultures. Errors bars are standard deviations.

IL22 has emerged as an important cytokine in the maintenance of epithelial cell homeostasis in mucosa tissues, including skin and gastrointestinal tract [16], [24]. In these tissues, IL22 mediates its function through IL22RA1, the membrane form of the IL22 receptor, which is predominantly expressed in mucosa epithelial cells [16], [24]. Thus, to determine if IL22 plays a regulatory role in HUC function, we first demonstrated that HUCs express IL22RA1. These cells also expressed low levels of the secreted soluble form of the IL22 receptor (IL22RA2, also known as the IL22 binding protein) (**Table 1**). To determine if HUCs could respond to IL22, we treated primary HUC cultures with a predetermined concentration of human recombinant IL22 (100 ng/mL) for 48 hours, and assessed the expression of CarAT, MR1 and two antimicrobial peptides that we found expressed in HUCs, S100A9 and lipocalin-2. Compared to untreated cultures, IL22 reduced expression of MR1 3.9-fold (or about 25% of the untreated cultures) (**Fig. 7**), while increasing S100A9 expression about 3-fold. IL22 moderately increased the expression of lipocalin-2 and CarAT (**Fig. 7**). This finding has important potential clinical significance as anti-muscarinic drugs are commonly used for treatment of certain human urinary disorders, such as urgency urinary incontinence and overactive bladder.

## Discussion

Human urothelial cells (HUCs) play an important role in the normal physiology, as well as the physiopathology, of the human bladder; however, information regarding HUC function is limited by the lack of an *in vitro* culture system that generates non-malignant HUC cultures. Here, we described a simple culture explant technique that reliably generates HUC cultures from human bladder biopsies that are free of fibroblast contamination. The biopsies were obtained after IRB approval from surgical patients who consented to urothelial biopsy. A small urothelial biopsy was collected at the end of the female patients’ indicated reconstructive pelvic surgery for pelvic organ prolapse and/or stress urinary incontinence. The biopsy is obtained during cystoscopy, a routine patient safety measure; clinically, the urothelium from these patients are considered grossly non-pathological, thus allowing us to interrogate normal functions of human urothelial cells. Furthermore, the generated cultures were free of fibroblasts, as verified by the high percentage of cells that co-express the epithelial cell-specific markers EpCAM and CD104. Since they consist primarily of urothelial cells, these cultures can be used to evaluate various *in vitro* functions of HUCs.

The simple explant technique described above does not require enzyme digestion of the bladder tissue, as described by others [25], [26]. We found that a single step incubation of the biopsy samples in culture media at 37°C facilitates removal of the upper urothelium layer from the underlying submucosa, which is the primary cellular source for fibroblast contamination. We conclude that the HUC cultures generated from bladder explant tissues are epithelial in nature, on the basis of cell morphology and expression of epithelial cell-specific surface molecules and intermediate filament keratins. Morphologically, our HUC cultures demonstrated the cobble-stone morphology characteristic of monolayer epithelial cell cultures. The HUCs were heterogeneous in size, comprised of islands of large cells surrounded by uniformly smaller cells. The clusters of larger cells contained many bi-nucleated cells, a cellular morphology typical of the superficial umbrella cells that line the lumen of the human bladder. We verified that the large bi-nucleated cells were umbrella cells by demonstrating that they express specific markers for umbrella cells: uroplakins (UPs) and cytokeratin 20 [4], [27]. Furthermore, TEM revealed that the bi-nucleated cells displayed uroplakin plaques (Uro Ps) and discoid fusiform vesicles (DFVs), both unique ultrastructures of umbrella cells [4].

Uro Ps are characteristic membrane structures composed of four UP types (Ia, Ib, II and III). UPIa is only expressed on the cell surface when it forms heterodimers with UPII [2]. When this complex is properly assembled, it is expressed on the cell surface where it serves as a receptor for UPEC via the adhesive FimH tip of UPEC’s type I pili, which selectively binds UPIa [19]–[21]. We demonstrated that UPEC adhered to HUCs in a FimH-dependent manner, indicating that UPs are functionally assembled and expressed on the HUC cell surface. We propose that these HUC cultures may be a useful tool to investigate the interactions between the human urothelium and UPEC, as well as the newly discovered urinary microbiota [11], [12].

That these cultures were indeed epithelial and free of fibroblast contamination was shown by their homogeneous co-expression of two epithelial cell-specific cell adhesion molecules: EpCAM and integrin-β4 (CD104). Expression of both cell surface markers also showed that cultured HUCs maintain expression of molecules that are critical for cell-cell interaction and intercellular structures. CD104 is important for forming hemi-desmosomes in urothelial cells of the basal layer, allowing them to anchor to the basal lamina [28]. EpCAM mediates homotypic cell adhesion through homodimerization, contributing to the formation of tight junctions through interaction with claudin 7 [29], [30]. We determined that cultured HUCs expressed claudin 7 (data not shown), indicating that they maintain their ability to form tight junctions. These cell-cell interactions at the monolayer cell level were revealed by TEM, which demonstrated that adjacent HUCs form lateral interdigitations with desmosomes. The expression of adhesion molecules, coupled to the existence of intercellular structures, indicates that the cultured HUCs maintain their ability to recapitulate the physical barrier function of urothelium *in vitro*. The HUCs generated from our explant technique are thus a potential source of cells to generate stratified human urothelium *in vitro*.

The cultured HUCs also uniformly express ICAM-1 (also known as CD54). It has been shown that expression of ICAM-1 is increased in bladder biopsies from patients with interstitial cystitis (IC), but undetectable in non-pathological bladder biopsies [31]. However, urothelial cells from IC patients express HLA-DR, while our cultured HUCs are HLA-DR negative. Whether the constitutive expression ICAM-1 is a unique feature of cultured HUCs remains to be determined.

In addition to the heterogeneity in cell size, cultured HUCs are also segregated into two distinct populations based on the expression of cell surface CD44. Expression of CD44 has been shown as a marker for basal urothelial cells; its expression is reduced in the intermediate layer and undetectable in the superficial umbrella cells [32], [33]. The two levels of CD44 expression suggest that HUCs in culture undergo differentiation from the less mature basal cells into the more differentiated and mature umbrella cells. The two populations of CD44-expressing HUCs also showed distinct patterns of expression of the Toll-like receptor 4 (TLR4) and its co-receptor CD14. While both CD44^lo^ and CD44^hi^ subpopulations expressed TLR4, only the CD44^hi^ HUCs expressed the CD14 co-receptor, suggesting that HUCs control signaling through TLR4 by regulating the expression of CD14.

The defense function of HUCs may be partially due to the co-expression of CD44 (which binds hyaluronic acid or HA) and TLR4/CD14 receptor complex (which responds to lipopolysaccharide of Gram-negative bacteria). Since CD44 facilitates the interaction of UPEC and urothelial cells in a murine model [34], the binding of HA to CD44 could function as a natural blocking ligand. Indeed, small case series report that intravesical HA treatment may have a role in reducing cystitis recurrence and mean UTI-recurrence time in humans [35], [36]. Thus, CD44/HA and TLR4/CD14 could collaborate to diminish bacterial infection. In addition to its regulatory role in the expression of S100A9 and lipocalin-2 as we reported here, whether IL22 also contributes to urothelium defense mechanisms against bacterial infection by regulating HUC production of HA or HUC expression of TLR4/CD14 remains to be elucidated.

The establishment of HUC cultures free of fibroblast contamination allows us to interrogate the potential functions that HUCs play in maintaining mucosa immunity of the human bladder. This clinically important aspect will advance our current knowledge deficit regarding the immune function of the human bladder, especially in the face of the emerging knowledge regarding the urinary microbiota [10]–[12]. For example, in the gastrointestinal tract, IL22 has emerged as an important regulatory cytokine. It maintains homeostasis in enterocytes and controls colonization of gut pathogens by modulating expression of antimicrobial peptides (AMPs), such as RegIIIγ, calprotectin, and lipocalin-2 [37], [38]. First, we showed that cultured HUCs express the membrane from of IL22R (IL22RA1). We then showed that IL22 increases expression of lipocalin-2 and the S100A9 subunit of calprotectin, but not the second subunit of calprotectin (S100A8) or RegIIIγ (data not shown). Selective expression of AMPs by HUCs may reflect the unique composition of tissue-specific microbiota, as well as a specific mechanism for controlling infections of tissue-specific pathogens. For example, RegIIIγ expression in enterocytes is induced by IL22 and is important for preventing *Candida albicans* infection in the gastrointestinal tract; UTI due to *C. albicans* has been described, but is a very uncommon lower urinary tract disorder [37], [39].

Lipocalin-2 has been shown to bind to a subset of iron chelators (siderophores) secreted by bacteria to obtain iron from the host [40]. The ability of siderophores to salvage host iron permits UPEC to grow in urine [41]. Sequestration of iron by siderophores is also required for UPEC to establish intracellular bacterial communities (IBC) in bladder urothelial cells. These IBCs have been associated with recurrent UTIs [42]. An IL22-mediated increase in lipocalin-2 expression would be expected to oppose bacterial salvage of host iron and thus inhibit UPEC growth in urine, UPEC invasion of HUCs, establishment of IBCs within HUCs, and recurrent UTIs.

In the absence of its subunit S100A8, S100A9 forms homodimers that can bind calcium. By sequestering calcium, S100A9 could control host-bacterial interactions that depend on calcium. S100A9 homodimers also interact with TLR4 to mediate an inflammatory response [43]. It is possible that, by modulating the expression of S100A9, IL22 could mediate an anti-inflammatory response through TLR4 in the HUC population that does not express the TLR4 co-receptor CD14.

Normal human bladder tissue is known to express both S100A9 and lipocalin-2; in bladder cancer, expression of both increases [44]. However, how their expression is regulated in health and diseases is not well understood; we provided the first direct evidence showing that IL22 regulates the expression of lipocalin-2 and S100A9 in primary HUCs express.

We also evaluated possible functions of IL22 in the physiopathology of lower urinary tract disorders. Bladder urothelium has recently been identified as a contributing factor in the development of overactive bladder. Increased release of acetylcholine by urothelium coupled with expression of muscarinic receptors by urothelial cells establishes a potential autocrine pathway [9]. It has been reported that human urothelial cells express CarAT, but not ChAT [6]–[8]. Indeed, our cultured HUCs expressed CarAT, but not ChAT. Furthermore, our cultured HUCs predominantly expressed MR1. We showed that IL22 potently inhibits MR1 expression, while only moderately increasing CarAT expression. IL22 could serve as a regulatory cytokine that contributes to the physiopathology of overactive bladder by modulating the acetylcholine response through inhibition MR1 expression. Whether IL22 also mediates a similar response in detrusor muscle cells remains to be determined.

While the cellular sources of IL22 in the human bladder have yet to be identified, it is known in other tissues that T cells and various innate cell types produce IL22 [24]. It is possible that HUCs can recruit these cells into the bladder submucosa and into urothelium itself. The high level of expression of the chemokine CCL20 by HUCs supports this notion. Indeed, we have preliminary TEM data showing that cells with lymphocyte morphology are present within the basal layer of urothelium (unpublished observation). Furthermore, HUCs also express IL7, IL15 and IL23, cytokines required for IL22-producing cell survival and expression of IL22.

## Materials and Methods

### Human Urothelial Cell Culture

The project was fully reviewed and approved by the Institutional review Board (RRB) for the protection of human subjects (Loyola University Health Science Division). Following IRB approval, and after written informed consent, a cystoscopically-directed biopsy was taken at the completion of reconstructive surgery in consenting adult women. The tissue was placed in saline and immediately transported to the laboratory. Bladder biopsy was transferred into culture media and incubated at 37°C for 2 hours to loosen the lamina propria. The culture media consists of a 3∶1 DMEM:Ham’s F12 medium with 5% FCS, 5.5 µg/mL (Life Technology), bovine insulin (Sigma-Aldrich), 0.4 µg/mL hydrocortisone (Invitrogen), 9.0 ng/mL cholera toxin (Sigma-Aldrich), 0.3% adenine hydrochloride, 1 mM sodium pyruvate, 10 ng/ml epidermal growth factor, 2.5 µg/ml amphotericin B, and 55 ng/ml gentamicin sulfate (Invitrogen). The urothelium layer was then separated from the lamina propria under dissecting microscope, cut into 1×1 cm^2^ fragments; the fragments were then anchored under coverslips to propagate HUCs. Tissue explants were removed after 1–2 weeks in culture and the proliferating cells were allowed to expand. The cells were then used or passage once and then used for experiments.

### Flow Cytometric Analysis

Harvested cells were washed once in RPMI-1640 media containing 2% FBS and 2–3×10^5^ cells were stained with a cocktail of fluorochrome-conjugated monoclonal antibodies for flow cytometric analysis using BD LSRFostessa; flow cytometric data were analyzed using Flowjo 7.5.5 (Treestar). All antibodies and their optimal dilutions were listed in Table 2.

**Table 2:** Antibodies Used in the Study.

Monoclonal antibodies	Clone	Sources	Dilutions
Anti-HLA ABC-PE	W6/32	eBioscience/Affymatrix	1∶5#
Anti-HLA DR-eF450	LN3	eBioscience/Affymatrix	1∶10
Anti-CD14-AF700	M5E2	Biolegends	1∶5
Anti-CD44-APC-eF780	IM7	eBioscience/Affymatrix	1∶40
Anti-CD54-FITC	HCD54	Biolegends	1∶5
Anti-CD284 (TLR4)-PE-Cy7	HTA125	eBioscience/Affymatrix	1∶5
Anti-CD104-eF660	439-98	eBioscience/Affymatrix	1∶10
Anti-CD326 (EpCam)-PerCp-eF710	1B7	eBioscience/Affymatrix	1∶10

# A volume of 5 µL of the indicated dilution was used.

### Transmission Electron Microscopy

Culture cells or bladder tissues were fixed in 4% glutaraldehyde in 0.1 M cacodylate buffer and then stained with 1% osmium in 0.1 M cacodylate buffer. Prepared cells or tissues were embedded in resin block, sectioned and analyzed using Hitachi- H600, 75 KV transmission electron microscope.

### Immunofluorescence assay

Briefly, 10^4^ primary HUCs in cultured medium were seeded into 24 well plates containing glass coverslips pre-treated with bovine plasma fibronectin (Sigma-Aldrich) and incubated at 37°C and 5% CO^2^ overnight. HUCs were then fixed in 3.7% formaldehyde in 0.1 M piperazine-N, N'bis (2-ethanesulfonic acid) PIPES buffer, pH 6.8 for 20 minutes at 4°C. Cells were then blocked and permeabilized with a blocking solution (10% normal goat serum, 0.1% triton X-100 in PBS) for 1 hour at room temperature and then stained with a mouse anti-human cytokeratin 20 (Abcam) and rabbit anti-bovine uroplakins (a gift from Dr. Tung-Tien Sun, NYU). After washing, cells were stained with a cocktail of goat anti-mouse IgG-FITC and donkey and donkey anti-rabbit IgG-PE (InVitrogen). To label actin, cells were stained with Cy5-labeled phalloidin at 1∶300 dilution in PBS for 30 minutes. Coverslips were then mounted onto glass slides with ProLong Gold Antifade containing DAPI (Invitrogen). Cells were examined using an epifluorescent DeltaVision microscope (Applied Precision) with a 60X objective lens and images were deconvolved with SoftWoRx software (Applied Precision).

### Quantitative RT-PCR

Total RNA was isolated using Trizol reagent (Sigma, Aldrich) according to manufacturer’s protocol. Isolated RNA was then treated with DNase using Ambion DNA-free kit and 0.5–2 µg of RNA was used for cDNA synthesis using the Invitrogen’s SuperScript II synthesis kit. Quantitative RT-PCR was performed on an Applied Biosystems 7100 as we have previously described [45]. Sequences for the primers are presented in Table 3; all are selected using Primer Express software (Aplied Biosystems). Samples were normalized to the housekeeping gene *Gapdh*. Levels of transcript were extrapolated from a standard curve constructed with five known concentrations ranging from 10 copies/µl to 100,000 copies/µl. Data were expressed as number of copies/µg RNA.

**Table 3:** Primer Sequences.

Genes	Forward	Reverse	Reference
*GAPDH*	GCACCGTCAAGGCTGAGAAC	CTTGTCATCAATGGAAATCCCATCACCATC	NM_008238.1
*CarAT*	GGACACAGTCAGCAACTTCAGC	CGGTTCACCTTGTCTTTGATGA	NM_000755.3
*MR1*	CGAAGTGGTGATCAAGATGCC	TGTTGTACGGTGTCCAGGTGA	NM_000738
*MR2*	CGACAGGTTGTTAGCGACATGA	TGTTCCCGATAATGGTCACCA	NM_000739
*IL7*	CAGACCAAGCGCAAAGTAGAAA	CGTAGTCATGATGACCGCAACT	GI:186363
*IL15*	CTTGCCATAGCCAGCTCTTCTT	CTGCACAAATCTATGGTTCCCA	GI:71680273
*IL23p19*	AGCTGTAATGCTGCTGTTGCTG	GGATCCTTTGCAAGCAGAACTG	GI:45768274
*CCL20*	TGTCAGTGCTGCTACTCCACCT	TCCATTCCAGAAAAGCCACAG	GI:18088856
*IL22RA1*	GCATGGAAGGTTCTGGCAA	AGGTACTGTGGTGTCCCTTCCT	NM_021258.3
*IL22RA2*	CAGTCAACGCATGAGTCTCTGA	CCTCCCGTAATAAGGTTCCTGT	NM_181309.1
*S100A8*	GGCCAAGCCTAACCGCTATAA	GACGTCTGCACCCTTTTTCCT	NM_002964.4
*S100A9*	GTGCAAGACGATGACTTGCAA	CAGCATGATGAACTCCTCGAAG	NM_002965.3
*Lipocalin-2*	CTGGATCAGGACTTTTGTTCCA	GGAAGACGATGTGGTTTTCAGG	NM_005564.3
*RegIIIγ*	CCTCCTCAAGTCGCAGACACTA	GCACAGACACCAGTTTTCCAGA	NM_001008387.2

### UPEC growth conditions

*E. coli* cystitis isolate NU14 and its isogenic *fimH* mutant NU14-1 [46] were grown under conditions known to induce type 1 piliation, namely serial subculture in static Luria Bertani broth for a total of 48 h at 37°C [47], [48]. Expression of type I pili by NU14, but not NU14-1, was confirmed by mannose-sensitive agglutination of 1% yeast in PBS [49].

### UPEC adherence assay

Bacterial adherence assays were performed, as previously described with modification [19]. Briefly, 10^4^ primary urothelial cells in TE medium were seeded into 24 well plates containing glass coverslips pre-treated with bovine plasma fibronectin (Sigma-Aldrich) and incubated at 37°C and 5% CO^2^ overnight. Prior to infection, the culture media was replaced with a medium consisting of RPMI, 5% FBS and Ham’s F-12. In duplicate wells, 10^5^ bacteria (NU14 or NU14-1) were added per well and the plate was centrifuged at 1400 rpm for 5 min to initiate bacteria contact with host cells. After 2 h of incubation at 37°C, the wells were washed five times with PBS to remove non-adherent bacteria and fixed in 3.7% formaldehyde in 0.1 M piperazine-N, N'bis (2-ethanesulfonic acid) PIPES buffer, pH 6.8 for 20 minutes at 4°C. Cells were blocked and permeabilized with block solution (10% normal goat serum, 0.1% triton X-100 in PBS) for 1 hour. The bacteria were stained with rabbit anti-*E. coli* antibodies (AbCam) followed by goat anti-rabbit Alexa Fluor 546 (Invitrogen) both diluted 1∶200 in block solution and incubated at 4°C for 1 h each. To label host actin, cells were stained with Cy5-labeled phalloidin at 1∶300 dilution in PBS for 30 minutes. Coverslips were then mounted onto glass slides with ProLong Gold Antifade containing DAPI (Invitrogen). Cells were examined using a DeltaVision microscope (Applied Precision) with a 60X objective lens and images were deconvolved with SoftWoRx software (Applied Precision). To quantify the number of urothelial cells with adherent bacteria, at least 200 urothelial cells were scored for the presence or absence of adherent bacteria. The percentage of urothelial cells with adherent UPEC was calculated by dividing the number of urothelial cells with bacteria by the total number of urothelial cells examined.
